# The Prevalence of Depression and Its Correlates Among Informal Caregivers of Stroke Patients in an Urban Community

**DOI:** 10.7759/cureus.51486

**Published:** 2024-01-01

**Authors:** Wan Mohd Aiman Wan Ab Rahman, Mazlina Mazlan

**Affiliations:** 1 Rehabilitation Medicine, Faculty of Medicine, Universiti Malaya, Kuala Lumpur, MYS; 2 Pediatrics, Universiti Sains Malaysia School of Medical Sciences, Kota Bharu, MYS; 3 Rehabilitation Medicine Unit, Universiti Sains Malaysia School of Medical Sciences, Kota Bharu, MYS

**Keywords:** family caregiver, stroke caregiver, stroke, caregiver burden, depression

## Abstract

Introduction

Stroke emerges as a prominent causative factor contributing to severe disability and functional limitations, necessitating a reliance on familial support for daily activities and participation in social engagements. Following hospital discharge, a considerable proportion of stroke survivors require comprehensive assistance, including physical, psychosocial, and financial support from caregivers within domestic settings. This transitional phase represents one of the most challenging periods for families. The reciprocal dynamics between stroke survivors and their caregivers are not only evident in the context of the caregiving process but also extend to their communal interactions. The disability and depressive symptomatology encountered by stroke survivors significantly impact the overall quality of life, both for the survivors themselves and for their caregivers. This study aims to determine the prevalence of depression among informal caregivers of stroke patients in Malaysia's urban setting, specifically in the University Malaya Medical Centre (UMMC), and examine the predicting associated factors and their relationship with the caregiver burden.

Methods

A cross-sectional study involving 54 informal caregivers was conducted via face-to-face and phone interviews with all informal caregivers of stroke patients who were attending outpatient specialist rehabilitation clinics, wards, and therapy areas at the UMMC. Patient Health Questionnaire-9 (PHQ-9) and Zarit Burden Interview-22 (ZBI-22) were used to determine the presence of depression and caregiver burden respectively. After conducting descriptive analysis, bivariate analysis was done using Chi-square, Fisher's exact, or Mann-Whitney U test. Multivariate analysis was then conducted using logistic regression.

Results

The overall prevalence of depression among informal caregivers of stroke patients was 18.5%. The prevalence of depression among female informal caregivers and caregivers who had been giving care for more than six months was 21% (n = 8) and 9.1% (n=3). Caregivers who provided care for more than six months, taking care of stroke patients who were diagnosed more than six months and had moderate to severe care burden were positively associated with depression.

Conclusion

Our study found the prevalence of depression among informal caregivers was high. Depression among caregivers was associated with giving care for more than six months, providing care for stroke patients who were diagnosed more than six months, and those with moderate to severe care burden. Therefore, screening for depression should focus on informal caregivers with patients using wheelchairs and higher care burdens.

## Introduction

The prevalence of stroke has been on the rise globally, contributing significantly to mortality and disability rates [1,2]. In Malaysia alone, there were 33,664 new cases of stroke recorded in 2016 [3,4], highlighting the growing incidence of this condition worldwide. Alarmingly, nearly half of stroke survivors worldwide require assistance with activities of daily living upon discharge from medical care [4]. Despite this, formal agencies dedicated to the care of stroke survivors are scarce in many countries, resulting in an overwhelming reliance on informal caregivers, often family members such as spouses, children, parents, or close friends, to provide the necessary support and care [5,6].

Depression among stroke caregivers is a common issue, and numerous studies have been conducted to assess its prevalence in this population [5,7-9]. Loh et al. [5] pointed out that the global prevalence of depression in this group stands at 40.2%. In another study by Berg Anu et al. [9], the incidence of depression ranged from 30% to 33%. Both of these studies highlight the reality of depression in stroke caregivers and the urgent need for addressing this concern.

Currently, there is just one study conducted by Azwanis et al. [10] that examines the prevalence of depression among informal stroke caregivers in Malaysia. This study was conducted in a rural setting in the east coast region of peninsular Malaysia and found a higher prevalence of depression at 63.8% compared to the global average. However, it's important to note that this study has limitations, particularly in its focus on a single predominant population group. Consequently, the findings cannot be generalized to the entire Malaysian population, as they exclusively represent the rural population, primarily comprising Malays, and do not reflect the diverse, multi-racial composition of Malaysia. Hence, there is a pressing need for another study to be conducted in an urban setting with a more diverse population to better understand the prevalence of depression among stroke caregivers in a multi-racial population.

Depression in caregivers of stroke survivors is closely associated with the level of caregiver burden, a connection that has been extensively explored in various studies [7,8,10]. Denno et al. [7] emphasized that as the caregiver burden escalates, the incidence of depression also rises. Supporting this correlation, a cross-sectional study by S. Achilike [8] further demonstrated a direct link between caregiver burden and depressive symptoms in stroke caregivers. Interestingly, at the local level, there has been a notable absence of research investigating the relationship between caregiver burden and depression among caregivers of stroke patients.

The objective of this study is to assess the prevalence of depression among informal caregivers of stroke patients in an urban community. Additionally, it seeks to investigate the factors associated with depression, whether they are related to the patient or caregiver, and to explore the connection between depression and caregiver burden. Understanding the risk factors and the interplay between caregiver burden and depression can offer valuable insights into identifying caregivers who may be susceptible to depression and facilitate the timely provision of intervention services.

## Materials and methods

A cross-sectional study was conducted at the Department of Rehabilitation Medicine, University Malaya Medical Centre (UMMC), Kuala Lumpur from March until August 2021. Medical ethics approvals were obtained from the Medical Research Ethics Committee, University of Malaya Medical Centre (MREC ID No: 202087-8967). All stroke patients who were still undergoing follow-up and attended the outpatient rehabilitation specialist clinic or outpatient therapy sessions were screened. Their primary caregivers which refer to the individuals whom being the most actively involved in helping the patients in daily life activities were approached and invited to participate in this study. Informed consent was taken before participation in this study.

The inclusion criteria for the caregivers were aged 18 years and above, family members or relatives of the patient, actively providing the caregiving (individual who provides most help in daily life activities if there were multiple caregivers), not receiving any stipend for caregiving, providing caregiving for at least one month, taking care a disable stroke patient (stroke patient who has limitation in daily life activities and social participation), and being able to read and write in Malay or English language. Caregivers who were unable to converse in Malay or English, diagnosed or undergoing treatment for any mental disorders, and paid caregivers of stroke patients were excluded. Eligible caregivers were either interviewed face to face or via a phone interview after they had consented. The questionnaire administered was either in English or Malay language based on the participant's preferences. Caregivers were selected using a convenience sampling technique, which was based on availability and willingness to contribute.

The questionnaires administered consist of four sections. The first section was information on the sociodemographic details of the caregivers including age, gender, race, religion, education level, care duration, care time, relationship with a stroke patient, receiving any financial aid assistance, employment status, and household income. The second section was information on the stroke patients including age, gender, race, religion, and stroke duration. The functional status of the patients was documented using both the Simplified Modified Rankin Scale Questionnaire (MRS) [11] and the Modified Barthel Index (MBI) [12].

The third section was information on the caregiver’s depressive symptoms. The Patient Healthcare Questionnaire-9 (PHQ-9) was used to assess depression. It assesses depressive symptoms and consists of nine items corresponding to the Diagnostic and Statistical Manual of Mental Disorders, Fifth Edition (DSM-5) diagnostic criteria for major depression. It is one of the most widely used depression screeners and is free and available in all major world languages. It is available in the Malay version and has been validated for use in a primary care setting in Malaysia [13]. It has demonstrated strong psychometric properties. Items are scored on a four-point Likert scale: 0 (“not at all"), 1 ("several days"), 2 ("more than half of the days"), or 3 ("nearly every day"). Scores ranging from 0 to 27 with higher scores indicating more severe depressive symptoms. Scores can be divided according to the level of severity: "minimal" (scores 0-4), "mild" (scores 5-9), "moderate" (scores 10-14), "moderately severe" (scores 15-19) and "severe" (scores 20-27), with cut-off scores determined from a general population. The score of the PHQ-9 (≥10) was used as the clinical cut-off for depression. A threshold score of 10 or higher has a sensitivity of 0.88 and a specificity of 0.85 for detecting depression [14]. A score of less than 10 will be regarded as no depression.

The final section was on the caregiver burden level using the Zarit Burden Care Interview (ZBI). The ZBI is a 22-item self-report measure of perceived caregiver burden. The score is graded on a 5-point Likert scale ranging from 0 to 4. The response options are 0 ("Never"), 1 ("Rarely"), 2 ("Sometimes"), 3 ("Frequently"), and 4 ("Nearly always"). The scores on the items are summed for the total score, with higher scores indicating a greater caregiver burden. The maximum total score is 88. It is available in the Malay version and has been validated in the Malaysian population as well [15]. ZBI total scores of 0 to 20 indicate no to minimal burden, scores of 21 to 40 indicate mild to moderate burden, and scores greater than 40 indicate moderate to severe burden.

Statistical analysis

All data were analyzed using the Statistical Package for the Social Sciences (IBM SPSS Statistics for Windows, IBM Corp., Version 26.0, Armonk, NY). The sociodemographic variables of caregivers, patients, and prevalence of depression were described using descriptive analysis. Bivariate analysis was conducted using chi-square, Fisher's exact, or Independent-samples Mann-Whitney U test to analyze the association between caregiver depression and the sociodemographic factors of caregivers and stroke patients. All variables with p<0.25 were further analyzed in multivariate logistic regression analysis to control for confounding. Variables with p-values less than 0.05 were considered to be statistically significant association with depression.

## Results

**Figure 1:**
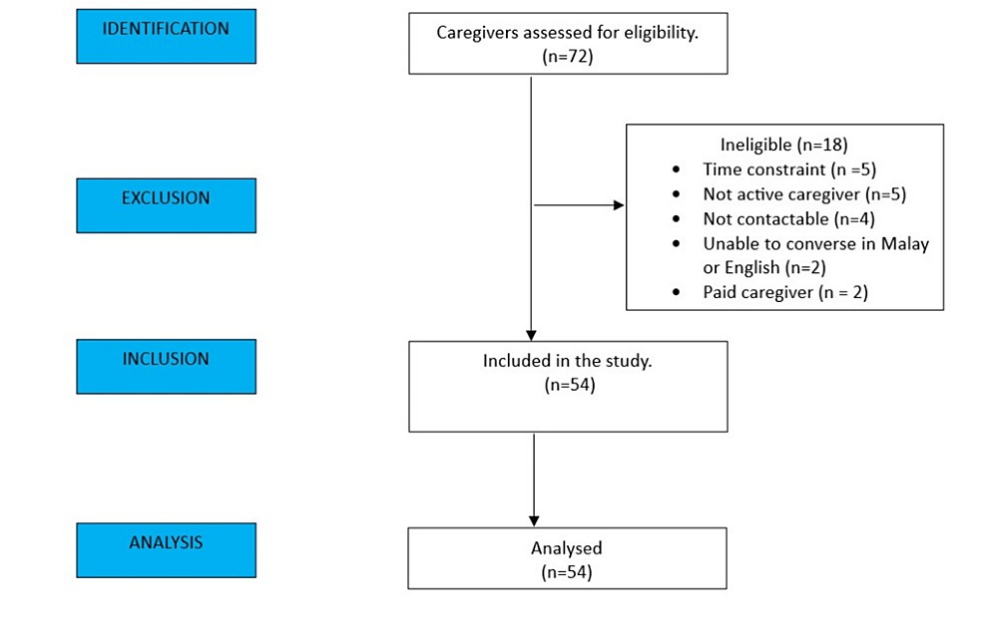
Study flow

Seventy-two stroke caregivers were approached from March 1 to August 31, 2021. Five caregivers declined to participate due to time constraints, five were not eligible as they were not active caregivers, four were not contactable due to the wrong contact number, two were unable to converse/write in Malay or English, and two were paid caregivers. A total of 54 caregivers were finally recruited into this study after they had consented (Figure 1).

Caregivers' profile

The mean age for caregivers was 48 ± 15.74 years old (Table 1). More than two-thirds of the caregivers were female (70.4%) and cared for the patients for more than six months duration (61.1%) for more than four hours per day (72.2%). Malay ethnicity comprises slightly less than half of the respondents (46.3%) followed by Indian and Chinese which are evenly distributed. As for the relationship between the caregivers and the stroke patients, spouses and parents make up the two most common relationships (44% and 39% respectively). A large proportion of the caregivers were from the lower socioeconomic group with a total household income of less than RM 4850. They are considered at the bottom 40% of the Malaysian income classification group [16]. Most of the caregivers did not receive any financial assistance; however, about 15% did receive financial aid either from social welfare, the Zakat Center, or the Social Security Scheme (SOCSO). The amount of financial aid was not specified.

**Table 1:** Sociodemographic characteristics of caregivers ZBI = Zarit Burden Interview

Variables (n= 54)	n (%)
Age, mean + SD, years	48.4+15.74
Gender	
Male	16 (29.6%)
Female	38 (70.4%)
Race	
Malay	25 (46.3%)
Chinese	15 (27.8%)
Indian	14 (25.9%)
Religion	
Islam	25 (46.3%)
Christian	8 (14.8%)
Buddha	7 (13%)
Hindu	14 (25.9%)
Education level	
Primary School	2 (3.7%)
Secondary School	25 (46.3%)
College/University	27 (50%)
Caregiving duration	
1-6 months	21 (38.9%)
>6 months	33 (61.1%)
Caregiving time/day	
< 4 hours	15 (27.8%)
≥4 hours	39 (72.2%)
Relationship	
Parents	4 (7.4%)
Spouse	24 (44.4%)
Child	21 (38.9%)
Siblings	3 (5.6%)
Others	2 (3.7%)
Financial aid	
Receive financial aid	8 (14.8%)
No financial aid	46 (85.2%)
Employment	
Unemployed	29 (53.7%)
Self-employed	10 (18.5%)
Full time employed	13 (24.1%)
Part-time employed	2 (3.7%)
Household income	
Below RM4850(B40)	38 (73.1%)
RM4850-RM10 959(M40)	10 (19.2%)
Above RM10959(T20)	4 (7.7%)
Care burden (ZBI-22)	
None or minimal care burden (0-20)	24 (44.4%)
Mild to moderate care burden (21-40)	22 (40.7%)
Moderate to severe care burden (>40)	8 (14.8%)

Stroke patients' profile

The mean age for the stroke patient was 60 ± 11.9 (Table 2). More than two-thirds of the patients were male (77.8%) and the duration of the stroke was more than six months. Based on the functional status, about one-third of the patients were moderately disabled. They may require some help, but they can walk without assistance. Only 11% of the stroke patients were totally dependent and required full assistance. For ambulation status, 64.8% did not require a wheelchair. Eighty-seven percent of the patients were still actively attending rehabilitation therapy programs.

**Table 2:** Sociodemographic characteristics of stroke patients

Variables (n= 54)	n (%)
Age, mean + SD, y	60.4+11.9
Gender	
Male	42 (77.8%)
Female	12 (22.2%)
Race	
Malay	25 (46.3%)
Chinese	15 (27.8%)
Indian	14 (25.9)
Religion	
Islam	25 (46.3%)
Christian	4 (7.4%)
Buddha	11 (20.4%)
Hindu	14 (25.9%)
Stroke duration	
1-6 months	20 (37%)
>6 months	34 (63%)
Modified Rankin Scale (MRS) level	
0	1 (1.9%)
1	10 (18.5%)
2	3 (5.6%)
3	20 (37%)
4	7 (13%)
5	13 (24.1%)
Modified Barthel Index (MBI)	
Total dependency (0-20)	6 (11.1%)
Severe dependency (21-60)	5 (9.3%)
Moderate dependency (61-90)	21 (38.9%)
Slight dependency (91-99)	11 (20.4%)
Independent (100)	11 (20.4%)
Ambulation assistance	
Wheelchair	19 (35.2%)
Non-wheelchair	35 (64.8%)
Attending therapy	
Yes	47 (87%)
No	7 (13%)

Depression prevalence and associated factors

The overall prevalence of depression among informal caregivers of stroke patients was 18.5% (n = 10). The prevalence of depression among female informal caregivers was 21% (n = 8). Of the informal caregivers who had been giving care for more than six months, 9.1% (n=3) were found to have depression. More than one-fifth (23.1%, n = 9) of the informal caregivers who were taken care of more than four hours had depression. In addition, 18.4% (n=7) of informal caregivers with income below RM 4850 were found to have depression. The prevalence of depression among informal caregivers who were taking care of stroke patients who were diagnosed more than six months and severely disabled (Modified Rankin Scale 5) was 8.8% (n=3) and 30.7% (n=4). Among informal caregivers of patients who required wheelchairs for ambulation and with moderate to severe care burden, 11.8% (n=4) and 62.5%(n=5), had depression.

From bivariate analysis, we found a statistically significant association between informal caregivers' depression and the care duration (p=0.03). In addition, the stroke duration was significantly associated with depression (p=0.03). Similarly, caregiver burden levels were also found to have a statistically significant association with caregiver depression (p=0.001). However, there was no statistically significant association between ambulation assistance and depression (p=0.14) (Table 3).

**Table 3:** Bivariate analysis of depression and associated factors

Characteristics (n= 54)	Depressed	Not depressed	p-value
	N (%)	N (%)	
Caregiver age			
mean + SD, y	43.67+20.95	49.38+14.57	0.356
Caregiver sex			0.705
Male	2 (25%)	14 (75%)	
Female	8 (21%)	30 (79%)	
Caregiver race			0.736
Malay	4 (16%)	21 (84%)	
Chinese	4 (26.7%)	11 (3.3%)	
Indian	2 (14.3%)	12 (85.7%)	
Caregiver religion			0.615
Islam	4 (16%)	21 (84%)	
Christian	3 (42.9%)	4 (57.1%)	
Buddha	1 (14.3%)	6 (85.7%)	
Hindu	2 (14.3%)	12 (85.7%)	
Education level			0.821
Primary school	0 (0%)	2 (100%)	
Secondary school	4 (16%)	21 (84%)	
College/University	6 (22.2%)	21 (77.8%)	
Care duration			0.035
1-6 months	7 (33.3%)	14 (66.7%)	
>6 months	3 (9.1%)	30 (91.9%)	
Care time			0.252
Less than 4 hours	1 (6.7%)	14 (93.3%)	
More than 4 hours	9 (23.1%)	30 (76.7%)	
Relationship			0.294
Parents	2 (50%)	2 (50%)	
Spouse	3 (12.5%)	21 (87.5%)	
Child	5 (29.4%)	16 (70.6%)	
Siblings	0 (0%)	3 (100%)	
Others	0 (0%)	2 (100%)	
Financial aid			0.326
Receive financial aid	0 (0%)	8 (100%)	
No financial aid	10 (21.7%)	36 (78.3%)	
Employment			1
Unemployed	6 (20.7%)	23 (79.3%)	
Self-employed	2 (20%)	8 (80%)	
Full-time employed	2 (15.4%)	11 (84.6%)	
Part-time employed	0 (0%)	2 (100%)	
Household income			1
Below RM4850(B40)	7 (18.4%)	31 (81.6%)	
BetweenRM4850 and RM10 959(M40)	2 (20%)	8 (80%)	
>Above RM10959(T20)	1 (25%)	3 (75%)	
Patient age			
Mean + SD, y	57.78+16.08	60.90+11.16	0.64
Patient sex			0.674
Male	7 (16.7%)	35 (83.3%)	
Female	3 (25%)	9 (75%)	
Patient race			0.7364
Malay	4 (16%)	21 (84%)	
Chinese	4 (26.7%)	11 (73.3%)	
Indian	2 (14.3%)	12 (85.7%)	
Patient religion			0.326
Islam	4 (16%)	21 (84%)	
Christian	0 (0%)	4 (100%)	
Buddha	4 (36.4%)	7 (63.4%)	
Hindu	2 (12.5%)	14 (87.5%)	
Patient stroke duration			0.028
1-6 months	7 (35%)	13 (65%)	
>6 months	3 (8.8%)	31 (91.2%)	
MRS level			0.439
0	0 (0%)	1 (100%)	
1	0 (0%)	10 (100%)	
2	0 (0%)	3 (100%)	
3	5 (25%)	15 (75%)	
4	1 (14.3%)	6 (85.7%)	
5	4 (30.7%)	9 (69.3%)	
MBI			0.685
Total dependency (0-20)	1 (16.7%)	5 (8.3%)	
Severe dependency (21-60)	1 (20%)	4 (80%)	
Moderate dependency (61-90)	6 (28.6%)	15 (71.4%)	
Slight dependency (91-99)	1 (9.1%)	10 (91.9%)	
Independent (100)	1 (9.1%)	10(91.9%)	
Ambulation assistance			0.139
Wheelchair	6 (31.6%)	13 (68.4%)	
Non-wheelchair	4 (11.8%)	31 (88.2%)	
Attending therapy			0.325
Yes	10 (21.3%)	37 (78.7%)	
No	0 (0%)	7 (100%)	
Care burden (ZBI-22)			0.001
None or minimal care burden (0-20)	1 (4.2%)	23 (95.8%)	
Mild to moderate care burden (21-40)	4 (18.2%)	18 (81.2%)	
Moderate to severe care burden (>40)	5 (62.5%)	3 (37.5%)	

From the multivariate logistic regression, only two factors were significantly predictive of informal caregiver depression: ambulation assistance and caregiver burden (Table 4). The caregiver for a stroke patient who does not require a wheelchair for ambulation is 10.6% less likely to have depression compared to the caregiver for a stroke patient who requires a wheelchair for ambulation (adjusted OR 0.106, 95% CI 0.011-0.978). The caregiver who has moderate to severe caregiver burden (ZBI score >40) is 35.4 times higher odds of having depression compared to the caregiver who has none or minimal caregiver burden (ZBI score 0-20) (adjusted OR 35.4, 95% CI 1.846-678.969).

**Table 4:** Multivariate logistics regression analysis for predicting depression among informal caregivers of stroke patients Adjusted R2 value = 0.551. Multiple logistic regression forward LR Model applied. Overall classified percentage = 88.9%, Hosmer and Lemeshow test, p = 0.492. No interactions were found between the variables (p= 0.227).

Independent variables	Wald statistics (df)	Adjusted odds ratio (95% CI)	p-value
Caregiving duration			
1 -6 months		1	
>6 months	2.031(1)	1.13 (0.021-60.86)	0.95
Stroke duration			
1-6 months		1	
>6 months	2.27(1)	0.031 (0-2.728)	0.129
Ambulation assistance			
Wheelchair		1	
None wheelchair	1.13(1)	0.106 (0.011-0.978)	0.048
Caregiver burden (ZBI-22)			
None or minimal care burden (0-20)	.(2)	1	0.022
Mild to moderate care burden (21-40)	1.35(1)	1.019 (0.072-14.52)	0.989
Moderate to severe care burden (>40)	1.51(1)	35.399 (1.846-678.969)	0.018

## Discussion

The present study strengthens our knowledge and understanding of factors associated with the increased risk of depression in the informal caregivers of stroke patients. In this cross-sectional study of informal caregivers of patients with stroke in an urban area in Malaysia, 18.5% had depression.

The prevalence is comparable to a recent local study conducted by Omar O et al. [17], which reported that 20.3% of the caregivers who were taking care of home-bound stroke patients in an urban community were depressed. However, the prevalence is much lower compared to another study performed locally by Azwanis et al. [10] in East Coast Malaysia which is considered a rural area and densely populated with a single ethnic population. The study reported a much higher prevalence of depression of 63.8%.

There could be a few reasons for the huge discrepancy in the prevalence rate although conducted locally in the Malaysian population. Malaysia is divided into West Malaysia and East Malaysia. West Malaysia is referring to Peninsular Malaysia and East Malaysia is referring to states in Borneo Island which are Sarawak and Sabah. All studies regarding the prevalence rate were conducted in Peninsular Malaysia. Peninsular Malaysia is further subdivided into west coast and east coast regions. The west coast region is more developed and populated compared to the east coast region. This has led to more concentrated healthcare services in the west coast region including rehabilitation services [18]. Therefore, stroke patients in west coast region can easily access rehabilitation services much earlier and this may explain the reason for the lower depression rate in studies conducted in urban areas compared to rural areas as the patients may have less disability as they undergo rehabilitation therapy.

A few studies have suggested that continuous support and help from specialists are necessary to reduce caregiving depression among family caregivers [19-21]. Having a support group also can make the caregiver feel empowered to alleviate the psychological stress and cope effectively thus reducing the care burden [22,23]. In our center, we have a regular support program for the caregivers and this could be one reason for the lower prevalence of depression.

Secondly, the disability level of a stroke patient may influence the rate of depressive symptoms. Findings from previous studies have concluded that severely functionally disabled stroke patients were positively related to caregiver depression [9,17,24]. However, in our study, disability level and independence level were not statistically significant. This could be due to a reduced number of patients who are severely or totally dependent on their daily activities. The proportion of severe and total dependency patients in our study is much lower (20%) compared to the study by Azwanis et al. which was at 39% [10]. Most of our patients require only minimal to moderate assistance including in ambulation. All the patients are undergoing rehabilitation therapy either as inpatient or outpatient and this has improved their independence.

In our study, we found that informal caregivers of stroke patients who required wheelchairs for ambulation are associated with higher odds of developing depression. This finding may reflect that stroke patients using wheelchairs may be more dependent on the caregivers especially when the patients are unable to self-propel the wheelchair and this indirectly adds to the caregiver burden.

Many studies have implied that a higher caregiver burden for stroke survivor caregivers is a known risk associated with caregiver depressive symptoms [8,25]. In the current study, we examined this association and found that 62.5% of informal caregivers with moderate to severe caregiver burden levels were depressed. The odds of informal caregivers with moderate to severe burden to have depression is 35 times compared to informal caregivers who have none or minimal care burden.

However, in the current study, we did not find any significant associations between depressive symptoms and ethnicity. A meta-analysis of 12 studies by Loh et al. [5] found that female and Caucasian race stroke caregivers were associated with a higher prevalence of depression. The studies included were mostly from the West with only two studies from Asian countries (Japan and South Korea).

Since we used a cross-sectional study design, it is difficult to establish a cause-effect relationship. The patient selection is also a limitation as we are using convenience sampling and due to the small sample size, the findings cannot be generalized to the whole population. Since the study was conducted during the COVID-19 pandemic, depression might be affected by COVID-19. However, in this study, we did not address the impact of COVID-19 on the informal caregiver’s depression. Nevertheless, the findings will help to inform practice and future research, specifically in Malaysia and Southeast Asia region.

## Conclusions

Our study showed that depression among informal caregivers of stroke patients in the urban population affects about one in five people. Depression is more prevalent in caregivers who taking care for more than six months, providing care for patients who had stroke for more than six months, and with moderate to severe burden. Therefore, screening informal caregivers of stroke patients for depression and treating them accordingly is crucial to decrease depression incidence. Special focus should be given to informal caregivers of stroke patients who require wheelchair assistance for ambulation and with moderate to severe burden.
